# Lived Experience Participation in Suicide Prevention Activities in Australia, a Scoping Review

**DOI:** 10.1111/hex.70241

**Published:** 2025-04-05

**Authors:** Hayley Purdon, Tania Pearce, Bess Jackson, Sarah Wayland, Myfanwy Maple

**Affiliations:** ^1^ The University of New England Armidale New South Wales Australia; ^2^ CQ University Sydney New South Wales Australia

**Keywords:** inclusion, lived experience, participation, suicide, suicide prevention

## Abstract

**Introduction:**

Lived experience inclusion is considered best practice in suicide prevention activities. Despite this, research remains limited exploring how individuals with lived experience actively engage in suicide research and prevention activities. The current scoping review aimed to map and summarise the existing literature describing the ‘how’ of lived experience participation in Australia.

**Methods:**

A scoping review according to the methodology of Arksey and O'Malley (2005) was undertaken with descriptive (e.g., study aims and methodology) and descriptive analytic data (such as key definitions and participation descriptors) being extracted from included studies.

**Results:**

A total of 42 studies met the inclusion criteria and were published between 2016 and 2023. There were many gaps in the data extracted, with participation descriptors and definitions often not reported in the literature. The included studies lacked clear and consistent definitions and practices when involving people with lived experience.

**Conclusion:**

Current processes for reporting experiences of lived experience participation in suicide prevention lack standardisation within peer‐reviewed publications. This review notes that there are gaps in the literature; however, the evidence base is growing for research that reports on suicide prevention research and activities that involve people with lived experience.

**Patient or Public Contribution:**

This study was created and undertaken by a PhD candidate with lived experience of thoughts of suicide, suicide attempt and caring for a loved one through suicide. A further team member has lived experience of being a suicide attempt survivor, continued thoughts of suicide and carer of family with suicidal thoughts. The study was informed by a Community Advisory Committee, of which four members have lived experience of suicide, with the remaining two having lived experience in areas where inclusion is paramount such as disability and suicide research. Ethics approval was not required for the participation of the Community Advisory Committee as they were providing advice only on the research conduct.

## 1 Introduction

In Australia, the inclusion of people with lived experience in suicide prevention was highlighted as an important focus, through the National Suicide Prevention Advisers Final Advice [1]. The report gave prominence to the inclusion of perspectives of personal experience of suicide in suicide prevention research, planning and policy. Despite this elevation, the literature base is sparse in the understanding of how people with lived experience use their perspectives in suicide prevention [2], or how they can be included in suicide prevention activities [3]. Understanding the details of participation of people with lived experience in suicide prevention is important to further the aims of inclusion.

In defining lived experience, academic literature utilises the catch‐all term *lived experience of suicide* to account for a range of experiences. Some research is specific to experience type such as suicide carer roles [4] whereas other research is broad in its definition to include mental health challenges alongside suicide [5]. In Australian suicide prevention practice, organisations engage with a broad view of the term. For example, Australia's peak body for suicide prevention, Suicide Prevention Australia, use the all‐encompassing definition of “touched by suicide in any way” [6]. Others utilise a nuanced view, such as the Black Dog Institute's Aboriginal and/or Torres Strait Islander definition that describes a holistic cultural view on social and emotional well‐being and suicide [7]. Roses in the Ocean, a lived experience of suicide organisation, specifies that lived experience of suicide is ‘having experienced suicidal thoughts, survived a suicide attempt, supported a loved one through suicidal crisis, or been bereaved by suicide’ [8]. Given the varying definitions of suicide, it is essential to clarify what experiences constitute a lived experience of suicide.

Lived experience inclusion is also identified in its role in research activities. In this space, people with personal experience of suicide have been included as research participants, to gain an understanding of the suicide experience such as states of mind preceding a near lethal suicide attempt [9] or how an individual is able to move from ideation‐to‐action [10]. A distinction has been drawn between participation as research subjects, and participation in research with various levels of control over the direction or outcomes of the research [11]. There has been pressure to expand involvement beyond research participation as research subjects. For example, the Lancet Psychiatry recently declared that a lack of inclusion beyond participation in research should be reported as a limitation of research seeking publication in the journal [12]. Research involving collaborative methods, such as co‐design, elevate the position of people with lived experience as partners in the research, but still retain them as participants in the research process [13]. Studies have explored how to best achieve lived experience inclusion in research by developing guiding principles [14, 15] although make no effort to map the scope of ways people with lived experience are involved.

From an analysis of the peer reviewed evidence, lived experience inclusion beyond participation in research is fragmented and narrow. Some areas of inclusion in suicide prevention have been studied, such as where people with lived experience are employed as peer workers [16, 17], storytellers [18], or involved in the delivery of an intervention such as Mental Health First Aid [19] and the Mates in Construction program [20]. Attempts have been made to generate frameworks or principles for inclusion in specific areas such as co‐creation [21] or inclusion in health‐care contexts [22]. There have been limited attempts to understand the ‘how’ of lived experience inclusion, detailing specifics around who is included, how they are included, and to what benefit or detriment.

While there have been few attempts to understand the specifics of lived experience inclusion, one notable example is Wayland et al. [2], who described the ways in which people with lived experience participate in suicide prevention generally. They found that people are often engaged to tell their lived experience story publicly, which may lead to a wearing down of resilience as participants relive events that are often traumatic. While evidence suggests that inclusion can enhance the effectiveness of interventions [23] or facilitate healing [2], inadequate support can lead to difficulties for individuals with lived experience working in the sector, as well as for those working alongside them due to the risk of vicarious trauma [24]. This highlights the need for a structured approach to understanding the safety needs of all involved in lived experience inclusion. Although a recent rapid review explored the involvement of people with lived experience in the development of interventions in suicide prevention in the last 10 years [3], there is a lack of evidence to show that inclusion of lived experience is undertaken in any systematic way [25]. To the best of our knowledge, no attempt has been made to systematically map the current literature on the lived experience inclusion in suicide prevention and identify any patterns in current practice.

The current review sought to better understand how people with lived experience of suicide participate in suicide prevention activities. The suicide experience and the way the suicide prevention sector responds is greatly impacted by social and cultural factors [26]. Even within defining accepted terminology in the field of suicidology, there have been reported differences between low/middle‐ and high‐income countries [27]. As the objective was exploratory in nature, a scoping review was appropriate [28] to obtain a broad view of the literature base. To minimise the impact of differing social and cultural contexts, as well as terminology used in the literature base, the review focussed on Australian‐specific studies only.

## 2 Methods

The scoping review was undertaken following the approach suggested by Arksey and O'Malley [28] and updated guidance from authors such as Peters et al. [29]. The following steps were undertaken: (1) identify the research question, (2) identify relevant studies, (3) study selection, (4) chart the data, (5) collate and summarise the results. The review approach was informed by the study's Community Advisory Committee through biannual meetings where the review plan and activities were presented and discussed by all members. Members had equal decision‐making power; however, in practice, Committee members offered suggestions which were then implemented by the lead author, with the lead author having final decision‐making power.

### 2.1 Stage 1: Review Questions

Through a review of the literature, apparent gaps were identified that, once filled, had the potential to create complete descriptions of how people with lived experience participate in suicide prevention. A draft protocol was developed and discussed with the study's Community Advisory Committee. It was decided that the review would seek to map the following aspects of lived experience inclusion in suicide prevention in Australia: (1) key definitions of lived experience inclusion, (2) pathways in and out of participation on suicide prevention, (3) specific roles of people with lived experience, and (4) impacts from involvement and safety measures employed.

### 2.2 Stage 2: Search Strategy

The Population, Concept and Context approach was used to guide the review and is presented in Table 1. The search was initially conducted on 11 June 2022. Due to delays in the review process, it was unable to be completed in a timely manner and therefore the search was further updated on 3 March 2023 and 25 November 2023. The search strategy was developed in conjunction with a discussion with the study Community Advisory Committee and a health librarian specialist. The search was pilot‐tested in Scopus using the following descriptors: ‘Liv* experience’ OR ‘direct experience’ OR peer OR consumer OR patient OR Widow OR ‘Exper* knowledge’ OR client OR survivor OR bereav* OR famil* OR loss OR ‘person* experience’ OR attempt* OR affect* OR care* OR grie* AND suicid* AND (prevent* OR interven* OR program* OR framework OR guide* OR research) AND Australia*. Three target articles were identified as being relevant for the review [2, 4, 32] and search results were checked for inclusion of these articles. Several terms were removed (e.g. client, consumer) as they included papers less likely to involve people with lived experience of suicide. Although searches were tested through Google for grey literature, the searches yielded extensive results and therefore grey literature was excluded from this study and will be reported elsewhere.

**Table 1:** Population, concept and context.

**Question: How do people with lived experience of suicide participate in suicide prevention in Australia?**	
**Population**	Lived experience is defined as ‘knowledge gained through direct, first‐hand involvement in everyday events, rather than through assumptions and constructs from other people, research, or media’ [30]. Therefore, as it applies to suicide, the study defines lived experience as people who have had first‐hand experience of suicide. This could be through experiencing thoughts of suicide, acts of suicide, caring for a person who is suicidal, being bereaved by suicide, experiencing suicide through work, or other experiences.
**Concept**	Participation is defined as any type of engagement in suicide prevention or research. Suicide prevention is any direct activity or document with the aim of reducing the impact of suicide. Suicide research is research that examines suicide or its prevention.
**Context**	The suicide experience and the way the suicide prevention sector responds are greatly impacted by social and cultural factors [31]. To ensure the study is feasible and most accurate for an Australian context, the project focusses on lived experience engagement in Australia only.

The final searches were conducted in Scopus, PsycINFO (ProQuest), PubMed and EBSCOhost. The final search strings are in Table 2.

**Table 2:** Search string by database.

Database	Search string
Scopus	TITLE‐ABS‐KEY ((suicide) AND (‘Liv* experience’ OR ‘direct experience’ OR peer OR ‘Exper* knowledge’ OR ‘person* experience’) AND (prevent* OR interven* OR program* OR framework OR guide* OR research OR resource) AND (participa* OR inclu* OR engage*)) AND (LIMIT‐TO (AFFILCOUNTRY, ‘Australia’))
PsycINFO	ab(suicide AND(‘Liv* experience’ OR ‘direct experience’ OR peer OR ‘Exper* knowledge’ OR ‘person* experience’) AND (prevent* OR interven* OR program* OR framework OR guide* OR research OR resource) AND (participa* OR inclu* OR engage*) AND Australia*) OR ti(suicide AND (‘Liv* experience’ OR ‘direct experience’ OR peer OR ‘Exper* knowledge’ OR ‘person* experience’) AND (prevent* OR interven* OR program* OR framework OR guide* OR research OR resource) AND (participa* OR inclu* OR engage*) AND Australia*)
PubMed	(suicide [Title/Abstract]) AND (‘Lived experience’[Title/Abstract] OR ‘direct experience’[Title/Abstract] OR peer[Title/Abstract] OR ‘Expert knowledge’[Title/Abstract] OR ‘personal experience’ [Title/Abstract]) AND (prevent*[Title/Abstract] OR interven*[Title/Abstract] OR program*[Title/Abstract] OR framework[Title/Abstract] OR guide*[Title/Abstract] OR research[Title/Abstract] OR resource[Title/Abstract]) AND (participa*[Title/Abstract] OR inclu*[Title/Abstract] OR engage* [Title/Abstract]) AND (Australia*[Title/Abstract])
EbscoHost	AB ((suicide) AND (‘Liv* experience’ OR ‘direct experience’ OR peer OR ‘Exper* knowledge’ OR ‘person* experience’) AND (prevent* OR interven* OR program* OR framework OR guide* OR research OR resource) AND (participa* OR inclu* OR engage*) AND Australia*) OR TI ((suicide) AND (‘Liv* experience’ OR ‘direct experience’ OR peer OR ‘Exper* knowledge’ OR ‘person* experience’) AND (prevent* OR interven* OR program* OR framework OR guide* OR research OR resource) AND (participa* OR inclu* OR engage*) AND Australia*)

### 2.3 Stage 3: Study Selection

To determine eligibility, a broad approach was taken to ensure a holistic picture of the literature was captured, and the research questions were answered. Peer‐reviewed original research, literature or case reviews, evaluations and discussion papers were included. Eligibility criteria were identified as: (1) papers that either include people with lived experience of suicide or describe how they can participate in suicide prevention activities, (2) papers that mention suicide specifically, although can also include topics related to mental health broadly, (3) papers that are focussed on the identification or testing of approaches for the prevention of suicide in Australia but may also consider international approaches (such as for comparison). This narrows the focus to papers where people with lived experience are included for the creation or evaluation of suicide prevention approaches, rather than inclusion as research subjects for the purpose of describing the suicide experience. Papers that include people with lived experience for the purpose of describing the suicide experience only, with no link to practice (e.g. psychological autopsy studies) were excluded. No publication date limit was applied to the search as it was suspected that the literature base would be small and recent.

The review sought to understand the participation of those with lived experience of suicide, in suicide prevention activities. Therefore, experiences of non‐suicidal self‐injury were not included in the review. As identified in attempts to define suicide [27, 33], the intention to cause death is conceptualised as a key distinction between suicidal and non‐suicidal self‐harm.

The search was conducted by the lead author. After removal of duplicates in Endnote, sources were uploaded to Covidence where the title and abstract was screened by two independent reviewers, with conflicts settled by a third reviewer. Full‐text review was conducted by two independent reviewers, with conflicts settled through discussion. Due to a high level of consensus at each stage of the review (77% consensus at title and abstract, 76% consensus at full‐text review), full‐text review was conducted by only the lead author for the second and third update of the search.

### 2.4 Stage 4: Charting Data

Following instructions by Arksey and O'Malley [28], data charting occurred in two steps. First, descriptive data such as aims of the study, methodology and key results were extracted. Second, descriptive analytical data were extracted such as descriptions of lived experience, methods of involvement and safety protocols employed by the studies. A data extraction template was developed in accordance with Arksey and O'Malley [28] and PRISMA guidelines [34], to which additional descriptive analytical items were added. To inform this process, the Community Advisory Committee was consulted (for data extraction instrument, see Table 3). Data extracted were checked by a member of the research team for accuracy and quality.

**Table 3:** Data extraction instrument.

Item	Description
#	Reference number allocated to resource.
Authors	Authors of the publication or document.
Title	Title of the resource.
Journal/source	Source of the publication or document.
Field of focus	Is the publication or document related to policy, research, or practice.
	Policy is defined as principles or guidance for the inclusion of people with lived experience. Research is defined as activity undertaken in a methodical/systematic manner to produce evidence or recommendations. Practice is defined as resources that are developed or implemented in the course of direct service provision.
Year of publication	Year the final resource was published.
Study population	Includes demographic information about participants, how many were included, etc.
Intervention details	What type of intervention or activity is the subject of the resource?
Study methodology	What formal or informal methods were used to develop the resource?
Representativeness	Are the findings representative of the Australian population?
Outcome measures	What measures were used in the analysis?
Findings	What are the high‐level findings or messages of the resource?
Practice implications	How can the suicide prevention sector respond to the findings?
Further research	What further research or action is suggested by the resource?
Definition of lived experience	How do the authors define lived experience?
Role of people with lived experience	What was the role of people with lived experience in the development of the resource?
	What activities were required of people with a lived experience?
	Were people with a lived experience given a different role to other participants?
Other parties involved	Who else was involved in the development of the resource (excluding authors)?
Framework for engagement	What frameworks were used to structure engagement with lived experience? What resources were developed to aid engagement (e.g., terms of reference, expressions of interest, etc.)?
Impact on people with lived experience	What were the impacts (positive or negative or neutral) on people with a lived experience who were engaged in the project?
Impact on project driver	What were the impacts (positive or negative or neutral) on the organisation, team or individual who drove the project?
How were people with lived experience recruited	How were people with lived experience recruited and selected for participation? What were the communication methods used to gather expressions of interest and how were they assessed?
	Were specific skills beyond their lived experience required?
Pathways	Were there any requirements before participation or pathways to continue participation after the project/resource development?
Payment of lived experience	Were people with a lived experience reimbursed for their time involved in the activity
Commitment	What time and/or resources required of the people with lived experience?
Safety	What structures or resources were supplied in supporting people with lived experience to participate in the activity? (e.g., access to support, trauma‐informed practice, etc.)
Follow up	What activities were conducted to support people with lived experience after the conclusion of the activity related to the resource

### 2.5 Stage 5: Collating, Summarising and Reporting Results

The extracted information was reviewed for descriptive themes according to Arksey and O'Malley [28]. Items recorded through the data extraction template were descriptively analysed in Microsoft Excel. This included counting the presence or absence of data extracted, as suggested by Pollock et al. [35]. The results are structured according to items extracted using the data extraction tool (Table 3), to answer the review questions outlined in Stage 1.

## 3 Results

**Figure 1:**
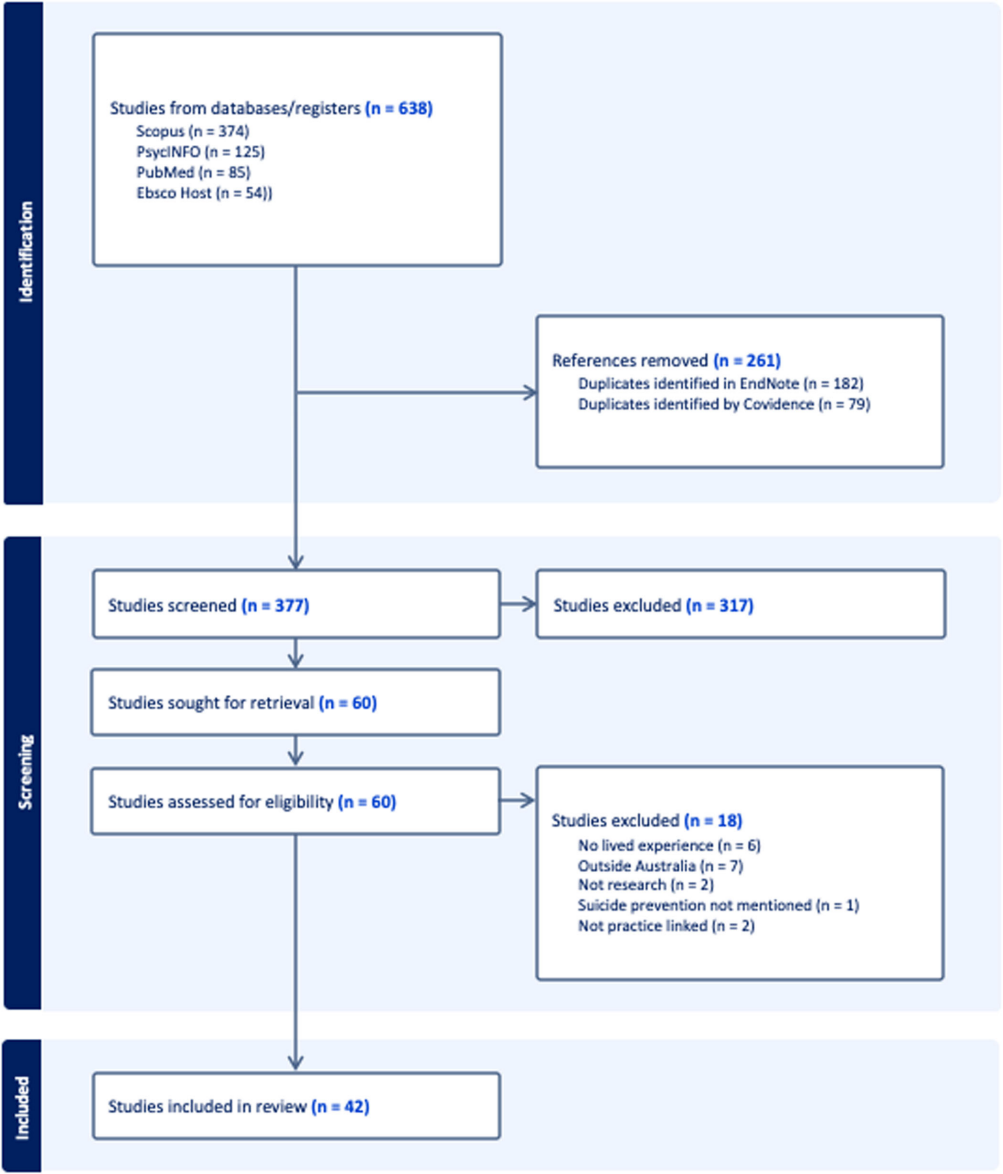
PRISMA flow diagram of included and excluded studies.

Following the searches of the databases, 638 papers were retrieved. After duplicates were removed and screening was conducted, 42 articles met inclusion criteria (Figure 1).

### 3.1 Characteristics of Sources of Evidence

Papers were published between 2016 and 2023 with 22 of the 42 included studies published since 2022.

**Figure 2:**
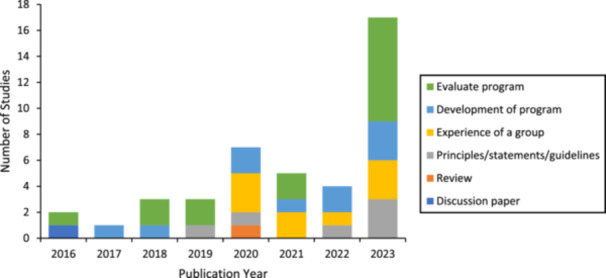
Included studies by publication year and purpose.

**Figure 3:**
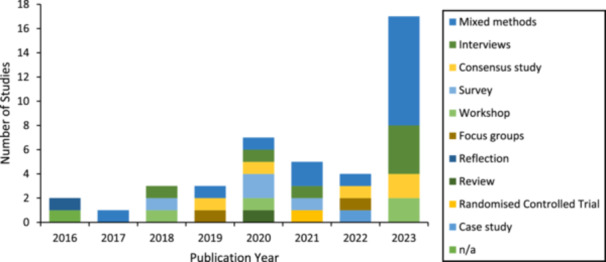
Included studies by year of publication and methodology.

**Figure 4:**
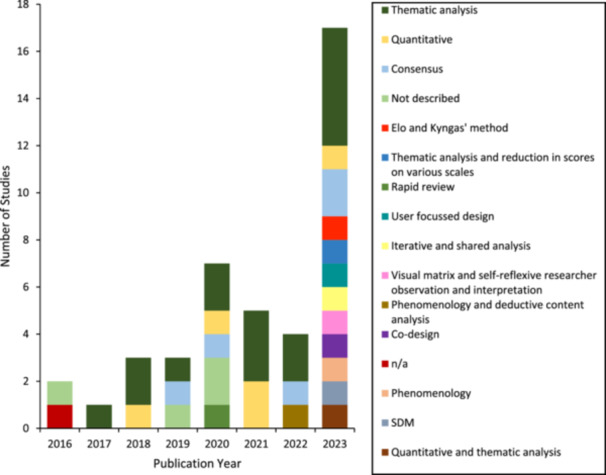
Included studies by year of publication and analysis methods.

The aims and methodologies of the included papers varied, and there was no clear pattern over time. The most frequent aim of the published research was the evaluation of a program (15 studies). The aims of included papers are presented in Figure 2. Methodologies employed by included studies were also varied with the most frequent methodology employed being mixed methods (15 studies). All methodologies are presented in Figure 3. Analysis methods are presented in Figure 4, with a diverse range of analytic methods employed in 2023. Thematic analysis was the most common analysis method (16 studies). Participant characteristics were not examined in the review as the review sought to understand the participation of people with lived experience across the research activity. The specific roles of people with lived experience are described below.

The descriptions of studies are reported in Table 4. Key descriptors for describing the ‘how’ of participation identified in the review are reported in Table 5.

**Table 4:** Summary of studies on lived experience inclusion in suicide prevention.

Title (Author, Year)	Intervention type and duration of intervention	Study populations	Aims and methodology of the study	Outcome measures and results (where relevant)
Confidence and attitudes of pharmacy students towards suicidal crises: patient simulation using people with a lived experience [36].	MHFA training involving lived experience enactments of experiencing mental health crisis.	186 final year Bachelor of Pharmacy students at The University of Sydney and 60 final year Master of Pharmacy Students.	Aim: Assess whether people with lived experience of mental illness as simulated patients impacted final year pharmacy students’ attitudes and confidence in providing care. Methodology: Survey examining confidence and attitude, analysed using parallel group repeated measures.	Using a one‐way ANOVA test, results show the involvement of people with a lived experience as simulated patients had a significant impact in improving and sustaining pharmacy students' confidence in providing MHFA for suicidal thoughts and behaviours. Impact of the simulation on attitudes to suicide was not clear.
Ethical Issues to Consider in Designing Suicide Prevention Studies: An Expert Consensus Study [14].	Not applicable, Delphi consensus study.	32 people with lived experience of suicide and 34 suicide prevention researchers.	Aim: To identify the most important ethical issues to consider when designing suicide prevention studies. Methodology: Consensus study with 80 statements rated from ‘essential’ to ‘should not be included’. Items were developed from a literature review.	There was significant agreement between and within the two groups of participants about the most highly rated statements. These included the importance of the ethical principles of merit, integrity, justice, and beneficence. Additionally, lived experience participants placed emphasis on the support and care for researchers, and suicide prevention researchers placed emphasis on the risk management protocols and support services for participants.
Acceptability, Safety, and Resonance of the Pilot Digital Suicide Prevention Campaign ‘Better Off With You’: Qualitative Study [37].	Better Off With You, digital suicide campaign underpinned by the Interpersonal Theory of Suicide.	13 people who had experienced suicidal thoughts and actions living within 2 PHN areas.	Aim: To explore the needs and preferences of people with lived experience of suicidal thoughts and actions to inform the development of Better Off With You. Methodology: Focus groups discussed campaign messaging, scope and approach. Creative agencies then created campaign collateral. User testing of campaign collateral was also conducted, and results analysed using thematic analysis.	The findings of this study highlight the challenges in developing a suicide prevention campaign that successfully conveys a clear, simple message. There was a strong preference to include first‐person perspectives in the campaign where stories are realistic and relatable while acknowledging the inequities of access to appropriate support.
A survey of people with lived experience of suicide‐related behaviour in Queensland, Australia: Their experiences with available resources [32].	Use of resources that provide information, support and guidance to people affected by suicide‐related behaviour.	175 participants who were self‐identifying as having attempted suicide (59), been bereaved by suicide (122), or cared for someone who had attempted or were bereaved by suicide (105).	Aim: To investigate the views about and experiences with support and resources of people with lived experience of suicide bereavement, suicide attempt, or caring. Methodology: Study was conducted using a survey.	People with lived experience participated through undertaking a survey. Most participants found resources helpful and user friendly and would recommend them to others. Unfortunately, a notable minority of survey respondents did not know that these resources existed or were unable to find the help they were looking for.
Crafting safe and effective suicide prevention media messages: outcomes from a workshop in Australia [38].	Use of media campaigns for suicide prevention.	21 participants in total (12 females and nine males). 9 participants were service providers, 6 were researchers, 3 were policy makers, and 2 were people with lived experience of being suicidal and/or being bereaved by suicide.	Aim: There were three aims of this study, to explore what suicide prevention experts consider to be essential characteristics of effective and safe suicide media campaigns, develop suicide prevention media messages and explore the impact that these messages might have on different audiences. Methodology: Data were gathered through workshop where participants discussed messages for three target audiences and was analysed using thematic analysis.	A message for each target audience was created. Different groups of participants placed emphasis or raised concerns about different elements of media messages.
Use of Web Conferencing Technology for Conducting Online Focus Groups Among Young People With Lived Experience of Suicidal Thoughts: Mixed Methods Research [39].	Web‐conferencing technology based online focus groups to determine feasibility.	The first research activity, a survey, obtained 40 participants aged between 16–25 years, 92.5% female. Other demographic information reported includes age, relationship status and living arrangements. All participants had lived experience of suicidal ideation.	Aim: To investigate feasibility of synchronous Web conferencing technology–based online focus groups (W‐OFGs) to engage young people with lived experience of suicidal thoughts in suicide prevention research. Methodology: Focus groups and pre‐ and post‐surveys. Researcher reflections were also included in results.	Findings from the study suggest that online focus groups are acceptable to young people in suicide prevention research. Findings also suggest that participants in a W‐OFG are less likely to elaborate on others' opinions as participants often used simple statements such as ‘I agree’, which can be simply substituted for nods in the conventional face‐to‐face focus group.
Preliminary Evaluation of Lived Experience of Suicide Training: Short‐, Medium‐ and Longer‐Term Impacts of Our Voices in Action Training [40].	Our voice in action (OVIA) us a two‐day introductory capacity building program for people with a lived experience of suicide delivered by facilitators with lived experience of suicide who have completed OVIA and undergone ‘Train the Trainer’ facilitator training.	89 people with lived experience of suicide who had completed OVIA workshops conducted between March 2018 and March 2020. Lived experiences of participants were have suicidal thoughts (60), have attempted suicide (41), being bereaved by suicide (63), have cared/continue to care for someone who is suicidal or attempted suicide (49), and two or more types of lived experience (68).	Aim: To assess the effectiveness of the OVIA program on learning outcomes, which included participant knowledge, attitudes, and self‐efficacy. Methodology: Pre‐ and post‐training survey, with 3 and 12 month follow up surveys.	Outcome indicators included domains of knowledge (suicide literacy and safe language when discussing suicide), attitudes to lived experience suicide prevention, and self‐efficacy (confidence in carrying out lived experience tasks and empowerment) and psychological distress. Findings support some hypothesised changes and that overall, the training program had positive impacts on participants and that the OVIA learning objectives are suitably matched with the desired outcomes.
Development of best practice guidelines for suicide‐related crisis response and aftercare in the emergency department or other acute settings: a Delphi expert consensus study [41].	Not applicable, Delphi review.	The study involved two panels consisting of Australian experts (39 health professionals, 50 consumers with lived experience). Panel composition varied slightly between rounds with roughly 60% of the lived experience panel having personal experience of suicidal behaviour, roughly 20% as a carer of someone with suicidal behaviour and roughly 20% with both personal experience and carer experience. The professional panel demographics did not report lived experience and listed the role of the person such as mental health nurse, social worker and academic. There were 8 professions listed.	Aim: Develop guidelines for staff responding to suicidal presentations in acute settings. Methodology: Delphi consensus study containing 525 items developed from systematic searches of academic and grey literature, as well as interviews from key informants.	A total of 420 items were rated as essential or important by at least 80% of both panels. The items included strategies that covered initial contact, assessment, referral, discharge and follow‐up, staff training, and linkage with community aftercare services. There were differences between the panels with some items rated higher by consumers and others by the professional panel.
Perspectives of rural health and human service practitioners following suicide prevention training programme in Australia: A thematic analysis [42].	A suicide prevention training program in regional (including rural and remote areas) South Australia which included involvement of a person with lived experience in the development and delivery of the training.	Participants were 248 health and human services workers. who had completed the full 1‐day training (engaging with people vulnerable to suicide) and lived and worked in South Australian regional communities. A subsample of 24 participants across eight sites was also interviewed. Professional backgrounds varied and included school counsellor and occupational therapist.	Aim: Explore the views of health and human services workers on a suicide prevention training programme in regional (including rural and remote areas) South Australia involving a person with lived experience in the development and delivery of the training. Methodology: Interviews were conducted through a formative dialogical evaluation methodology using open ended questions. Transcripts were thematically analysed.	Five themes were identified; Coproduction is key, It is okay to ask the question, Caring for my community, I can make a difference and Learning for future training. The overall meta‐theme was ‘Involvement of a person with lived experience in suicide prevention training supports regional communities to look out for people at risk of suicide’.
A Mobile Text Message Intervention to Reduce Repeat Suicidal Episodes: Design and Development of Reconnecting after a Suicide Attempt (RAFT) [43].	The aim of RAFT is to provide a text message–based follow‐up intervention, combining regular SMS contacts and links to Web‐based therapeutic content and resources focused on the 6 content areas.	The study engaged a lived experience design group, Centre for Research Excellence in Suicide Prevention's Lived Experience Committee (CRESP LEC), the Black Dog Institute's Lived Experience Advisory Panel (BDI LEAP) and clinical design group. For the lived experience design group, the 7 participants had a history of a suicide attempt, but not in the immediately preceding month, and were not currently experiencing severe suicidal ideation (if a current suicide plan, means, or intent was endorsed). The paper does not report on the composition of the CRESP LEC, BDI LEAP and clinical design group.	Aim: Design of a brief Web‐based intervention targeting proximal risk factors and the needs of this population during the post‐attempt period. Methodology: Data were collected using focus groups and analysed using thematic analysis. The intervention design was created by the research team and reviewed by the clinical design team. After designed by a creative agency, lived experience members and clinical members reviewed the end product.	The study informed the development and design of the RAFT SMS‐based brief contact intervention. Common features identified in the study include the need for proactive follow‐up immediately following discharge from the ED, with messages of support and encouragement with relevant support contacts.
Developing best practice guidelines for the psychosocial assessment of Aboriginal and Torres Strait Islander people presenting to hospital with self‐harm and suicidal thoughts [44].	Development of a set of underlying principles of culturally competent practice and recommendations for processes of effective and appropriate engagement; risks, needs and strengths to be assessed; formulation of psychosocial assessment; and recommendations specific to children and young people.	An expert panel comprising 28 individuals with clinical, community‐based and lived experience in Aboriginal and Torres Strait Islander mental health and/or suicide prevention.	Aim: To develop guidelines for culturally responsive psychosocial assessment of Aboriginal and Torres Strait Islander people presenting to hospital with self‐harm and suicidal thoughts. Methodology: Delphi consensus containing 286 items developed through systematic search and review of research literature, existing guidelines and grey literature.	A total of 226 statements reached consensus and were endorsed by ⩾ 90% of panellists. These were included in the guidelines document produced from the study.
Involving mental health service users in suicide‐related research: A qualitative enquiry model [45].	The paper describes the approach taken by the research team in undertaking first person interviews with people who experience suicidality.	This paper describes a research approach and does not describe details of conducting the research. Therefore, participants are not described in detail. Generally, the research included executive, clinical, and service user representatives of the public mental health service, senior representatives of the peak body for suicide prevention in Australia, the relevant health and medical human research ethics committee, and mental health nursing academics at three universities. The group who were participants of the study is not identified.	Aim: The paper describes the process of developing and deploying a research model as part of a multi‐method qualitative study investigating suicidal service‐users' experiences of mental health nursing care. Methodology: The paper reflects on conducting semi‐structured interviews with service users but does not specify formal methods for the creation of knowledge in the paper.	The paper outlines reflections from the research team practice and a framework for better engagement. It was identified that service user participation should consider: Researcher competency, participant recruitment, consent and confidentiality, support and protection of participants.
Carers' motivations for, and experiences of, participating in suicide research [4].	A survey on the experiences of self‐identified carers of people who had previously attempted suicide. This publication focusses on why this group participate in research.	758 individuals who self‐identified as being carers for individuals who had previously attempted suicide completed the survey. The paper reported on the participant's relationship to the person who had attempted suicide with responses including child, friend and partner.	Aim: Explore what motivates people to participate in suicide research and how they experienced participating in the current study. Methodology: An online survey was conducted with 54 questions. This paper is focussed on two open‐ended questions within the survey. Data were analysed using thematic analysis.	Regarding what motivates people to participate in suicide research, three themes were identified, namely that good data can be a powerful tool for change, participation was an attempt to ensure inclusion of lived experience, and participants were motivated to effect change in the mental health system broadly, or to shift workforce views on responding to attempts. Regarding how people experienced participation in the study, four themes were identified namely that involvement was important, despite the fact that it elicited difficult emotions, involvement triggered painful memories and experiences, and participation enabled safe reflecting on the process of providing care post suicide attempt, via survey participation. Finally, the final theme contained outlier sub‐themes.
Assessing students' mental health crisis skills via consumers with lived experience: a qualitative evaluation [46].	After completing MHFA training students participated in simulated assessments of their ability to provide first aid. Students were either randomly allocated to participate in (n 1⁄4 40) or observe (n 1⁄4 146) a simulated scenario of a person experiencing symptoms of a mental health crisis in a community pharmacy.	22 final year Bachelor of Pharmacy students at The University of Sydney who had completed MHFA training as a part of the curriculum and participated as observer or participant in at least one simulated scenario of a person experiencing symptoms of a mental health crisis in a community pharmacy.	Aim: To explore the perceived benefits, for both students and simulated patients, of assessing MHFA skills (post‐training) through simulated patient role‐plays of mental health crises. Methodology: Focus groups using semi‐structured interview guides. Thematic analysis was used to identify themes.	Five themes were identified and were the benefits to students and simulated patients, the value of lived experience, challenges with suicide assessment, confidence in communicating with people experiencing mental health problems or crises and the value of immediate feedback and debrief.
Drafting the Aboriginal and Islander Mental Health Initiative for Youth (AIMhi‐Y) App: Results of a formative mixed methods study [47].	AIMhi‐ Y App draft, which is a strengths‐based early intervention wellbeing app for Aboriginal and Torres Strait Islander youth.	Co‐design workshops: 45 Aboriginal and Torres Strait Islander youth, aged 10–18 years, from three sites in the Northern Territory (NT). Online survey: 75 Aboriginal and Torres Strait Islander people. Although not specific with numbers, it was stated that psychological distress was a common experience for youth in this study and many participants across all groups, had recently experienced bereavement as a result of death by suicide. One participant in this study expressed current suicidal ideation.	Aim: Development of the AIMhi‐Y App which engaged Aboriginal and Torres Strait Islander youth. Methodology: Participatory design research approach co‐design workshops across three sites with five groups of young people. The study also included a peer‐supported online survey exploring topics around acceptability.	The study identified that participants faced barriers to help‐seeking despite experiencing psychological distress. Apps were perceived as a potential solution to overcome barriers, and preferred app characteristics included a strength‐based approach, mental health information, relatable content, and a fun, appealing, easy‐to‐use interface that encouraged app progression.
Consumer and carer perspectives of a zero suicide prevention program: A qualitative study [48].	Suicide Prevention Pathway (SPP) is a suicide aftercare program comprised of seven elements: screening, assessment, risk formulation, safety planning, preventing access to lethal means, structured follow‐up, and transition, for at‐risk individuals seeking care, and mandates the development and routine revision of a tailored safety plan in a collaborative, evolving process.	10 consumers and 5 carers who had experienced placement on a Suicide Prevention Pathway based on the Zero Suicide framework.	Aim: Examine the experiences and perspectives of consumers who have been placed on the SPP, and their carers. Methodology: Semi‐structured interviews were conducted, and data analysed using a constructivist grounded theory approach and a generic inductive thematic analysis where relevant.	Three themes were identified, feeling safe and valued, intersection of consumer and staff/organisational needs, and the importance of the ‘whole picture’. Overall, consumers and their carers reported a favourable experience of the Suicide Prevention Pathway; however, there were several areas identified for improvement.
A lived experience co‐designed study protocol for a randomised control trial: the Attempted Suicide Short Intervention Program (ASSIP) or Brief Cognitive Behavioural Therapy as additional interventions after a suicide attempt compared to a standard Suicide Prevention Pathway (SPP) [68].	Study protocol for an RCT involving Attempted Suicide Short Intervention Program (ASSIP) and Brief Cognitive Behavioural Therapy (CBT) for Suicide Prevention and Suicide Prevention Pathway (standard care approach).	The paper describes a research protocol. People who are 16 and over, attempt suicide or experience suicidality after a suicide attempt, present to the Gold Coast Mental Health and Specialist Services, are placed on the Suicide Prevention Pathway (SPP), and meet the eligibility criteria, were offered the opportunity to participate. A sample size of 137 participants was sought.	Aims of proposed study: Assessing the addition of two structured psychological interventions to treatment as usual under the ASSIP and Brief CBT interventions and providing a cost‐ benefit analysis of the interventions. Methodology of proposed study: Randomised controlled trial with blinding of those assessing the outcomes.	The proposed study will measure representation to hospitals with suicide attempts, time to representation to hospital with a suicide attempt and proportion representing within 7, 14, 30, and 90 days. Suicide ideation and death by suicide rates will also be examined. Measures include self‐reported levels of suicidality, depression, anxiety, stress, resilience, problem‐solving skills, and self‐ and therapist‐reported level of therapeutic engagement are measured.
Taking the next step: A qualitative study examining processes of change in a suicide prevention program incorporating peer‐workers [17].	Next Steps is a follow‐up aftercare service for people over the age of 16 who have presented to local Emergency Departments (ED) following a suicide attempt or because of high risk for suicide. The intervention is based in the Illawarra Shoalhaven region on the south‐east coast of New South Wales, Australia.	6 peer‐workers and 5 clinicians (*n* = 11) employed in the suicide prevention aftercare program Next Steps (55% female). Mental health clinicians in this study were registered psychologists who met national accreditation standards with the Australian Health Practitioner Regulation Agency. Peer workers had received 2‐day training course related to the Next Steps program. The 11 participants represent 100% of the clinicians from the Next Steps Program, and 75% of the peer‐workers from the program.	Aim: To examine the processes facilitating change in an aftercare suicide prevention program featuring peer‐workers from the perspective of clinicians and peer‐workers employed in the service. Methodology: Online survey and telephone interviews collecting qualitative data which were analysed using thematic analysis under a constructionist lens, co‐created by researcher and participant.	The study identified four themes for promoting change, utilising lived experience, emotional availability of peers, building lives worth living, and consumer driven care. A less direct mechanism of change was also identified, consultation in the context of risk.
Developing an Intervention for Suicide Prevention: A Rapid Review of Lived Experience Involvement [3].	Not applicable, rapid review.	Not applicable, rapid review.	Aim: To synthesise available studies using lived experience of suicidality to guide the development of suicide prevention interventions. Methodology: Rapid review following PRISMA guidelines of the literature from 2010 to 2019.	The study identified that focus groups and thematic analysis were common methods for understanding the suicide experience. The studies identified focused on participant preferences, experiences, and recommendations for suicide prevention interventions. Translation from research findings into a physical intervention was described in limited detail across all studies.
How is participating in suicide prevention activities experienced by those with lived and living experiences of suicide in Australia? A qualitative study [2].	Experiences of people with lived experience of suicide who participate in suicide prevention.	20 participants who were lived experience representatives within suicide prevention (70% female). To be eligible to participate, individuals were required to be 18 years of age and over, have a self‐reported lived experience of suicide (their own prior suicide attempt (10), caring for someone who has made a suicide attempt or is suicidal (number not reported), or bereaved by suicide(13)), have previously undertaken speaker training (self‐defined as having participated in training in how to tell of their lived experience), been engaged in voluntarily speaking about their lived experience for more than 12 months, and located in Australia.	Aim: To describe how authentic inclusion of lived experience is experienced by those who have lived experience and participate in suicide prevention activities in Australia. Methodology: Qualitative narrative enquiry with data analysed using thematic analysis.	The findings demonstrated a broad range of experiences in both time since speaker training and active participation in the suicide prevention field, reflections on the definition of lived experience, and the scope of the speaking opportunities available. Themes identified were, definitional challenges and a lack of consensus, awareness of the benefits from lived experience participation, challenges that stem from lived experience involvement and the practical and emotional labour of speaking.
Ethical and political implications of the turn to stories in suicide prevention [49].	Not applicable, philosophical discussion paper.	Not applicable, philosophical discussion paper.	Aim: Paper argues that organisations make possible but shape personal stories of suicide and therefore shape public meanings of suicide. Methodology: Critical argument drawing on literature and methods of narrative including narrative approaches to bioethics.	The paper argued for the importance of personal stories of suicide for meaning‐making, power, and social identity. It also argued that they reproduce and normalise particular ways of thinking, acting, and communicating that reinforce the institutional logics of suicidology. It calls for deeper examination of the social contexts in which stories are told.
Developing a Suicide Prevention Social Media Campaign With Young People (The #Chatsafe Project): co‐design Approach [13].	Co‐design workshops to design #chatsafe suicide prevention social media campaign.	134 young people aged between 17 and 25 years. Lived experience was captured with 103 reporting suicidal ideation, 112 reported supporting a friend experiencing suicidal ideation and 59 reporting losing someone close to suicide.	Aim: To document key elements of the co‐design process; to evaluate young people's experiences of the co‐design process; and to capture young people's recommendations for the #chatsafe suicide prevention social media campaign. Methodology: Participatory co‐design process to generate recommendations for a web‐based communication about suicide.	The study reported that participants viewed the co‐design workshops as safe and enjoyable. Outcomes included feeling better equipped to communicate safely about suicide on the web and feeling better able to identify and support others who may be at risk of suicide. Key recommendations for the campaign strategy were that young people wanted to see bite‐sized sections of the guidelines come to life via shareable content such as short videos, animations, photographs, and images. They wanted to feel visible in campaign materials and wanted all materials to be fully inclusive and linked to resources and support services.
Developing a post‐discharge suicide prevention intervention for children and young people: a qualitative study of integrating the lived‐experience of young people, their carers, and mental health clinicians [50].	A post‐discharge suicide prevention intervention for children and young people delivered by phone.	Participants were 5 young people (aged 17–25 years) with lived‐experience of discharge from the Queensland Children's Hospital emergency department after a suicide attempt or self‐harm incident (3 male), 3 females with lived experience caring for a young person who had been discharged from the emergency following a suicide attempt or self‐harm incident, and 10 mental health clinician working in the emergency department (6 female).	Aim: To integrate lived experience into the design of a suicide prevention intervention delivered by phone to young people post‐discharge from an emergency department (ED) for suicide risk or self‐harm. Methodology: Focus groups, phenomenological analysis and deductive content analysis.	The study identified that follow up phone interventions needed to consider person‐centred focus, the phone‐call dynamic, and the phone‐call purpose. Participants also preferred an intervention that was structured, consistent, finite, authentic, able to facilitate and empower growing independence, and achievable of young people after an ED presentation was desired. These themes resulted in the design of an intervention in alignment with the themes.
Exploring Community‐Based Suicide Prevention in the Context of Rural Australia: A Qualitative Study [51].	Descriptions of community‐based suicide prevention in the context of rural Australia.	37 participants (lived experience 48.6%) who were self‐identified experts, working in rural community‐based suicide prevention (community services, program providers, research, and policy development) around Australia. Specific lived experience was not identified.	Aim: To explore community‐based suicide prevention. Methodology: Focus groups and semi‐structured interviews with data analysed using thematic analysis.	The study identified three themes relating to community‐based suicide prevention, community led initiatives, meeting community needs, and programs to improve health and suicidality. Implementing community‐based suicide prevention needs community‐level engagement and partnerships. Definitional challenges were identified, as was a preference for nonclinical supports.
Stakeholder insights into implementing a systems‐based suicide prevention program in regional and rural Tasmanian communities [52].	Lifespan suicide prevention trials in Tasmanian communities.	46 participants comprising Trial Site Working Group members (n = 25), Tasmania's Primary Health Network employees (*n* = 7), and other key stakeholders (*n* = 14). Approximately half of participants had a lived experience of suicide.	Aim: To explore key stakeholder perceptions of implementing a systems‐based suicide prevention program in regional and rural communities in Tasmania, Australia. Methodology: Participatory Action Research design using focus groups, interviews and observational data. Data analysed using thematic analysis.	The study identified themes including how the Trial was established in Tasmania, Working Group governance structures and processes, communication and engagement processes, reaching priority population groups, the LifeSpan model and activity development, and the effectiveness, reach and sustainability of activities.
A model of lived experience leadership for transformative systems change: Activating Lived Experience Leadership (ALEL) project [5].	Presents a model for recognition and understanding of lived experience leadership in mental health and social sectors. Also outlines a PAR process in great detail for the generation of the framework.	Lived experience leaders and sector leaders (not stated if these have lived experience or not), 31 participants for focus groups, 14 participants in interviews and 48 responses to survey. Specific details are reported elsewhere in grey literature sources.	Aim: Describe a lived experience leadership model, developed as part of the Activating Lived Experience Leadership (ALEL). Methodology: Focus groups and a national survey with data interpreted through an iterative and shared analysis. Other methods included a project advisory group, information literacy workshops, a community of practice group and two Systems and Sector Leaders' Summits.	The paper describes a model framing lived experience leadership as a social movement for recognition, inclusion and justice and is composed of six leadership actions: centres lived experience; stands up and speaks out; champions justice; nurtures connected and collective spaces; mobilises strategically; and leads change. Leadership is also guided by the values of integrity, authenticity, mutuality and intersectionality, and the key positionings of staying peer and sharing power.
Understanding and detecting behaviours before a suicide attempt: A mixed‐methods study [53].	Three studies to test the acceptability and feasibility of a computer vision algorithm to identify crisis behaviours.	Study 1: representative sample of Australian population with about a third having lived experience (346 of a total of 1090), interviews with first responders and people with lived experience of suicide were also conducted and the results of which will be reported elsewhere. Study 2 was analysis of CCTV footage; Study 3 developed an algorithm.	Aim: To examine the acceptability and feasibility of using an automated computer system to identify crisis behaviours. Methodology: Mixed methods study using acceptability survey, manual structured analysis of closed‐circuit television footage and configuration of a computer vision algorithm.	The study identified that attitudes were positive towards research using closed‐circuit television and artificial intelligence for suicide prevention, including among those with lived experience. The second study revealed that there are identifiable behaviours, including repetitive pacing and an extended stay. Finally, the automated behaviour recognition algorithm was able to correctly identify 80% of acted crisis clips and correctly reject 90% of acted noncrisis clips.
Co‐creation of new knowledge: Good fortune or good management? [54].	Suicide aftercare program called ‘Eclipse’. Details of intervention not described in paper. Paper describes case study of the development of the intervention and application of co‐partnerships model.	The overall sample (*n* = 11) consisted of three different groups of participants: researchers (*n* = 3), TSO stakeholders, including peer workers with lived experience (*n* = 5) and funders (*n* = 3). Further details are not reported.	Aim: To document and describe the events and critical factors influencing the implementation of co‐creation, including the value of co‐creation opportunities presented, to explore the perspectives of primary stakeholders, including researchers, to illustrate their understanding of the implementation of co‐creation, and to revisit the proposed model and make any adjustments. Methodology: Paper presents a case study where thematic description of key elements of the coproduction framework were analysed.	The study identified three themes, understanding the language and practice of co‐creation, perception of trust formation, and the value of co‐creation opportunities. Ultimately, implementing co‐creation with or between researchers, industry and people with lived experience requires trust, reciprocity, good fortune, and good management. While implementing co‐creation, the co‐creation framework was revised to include additional elements identified as missing from the initially proposed framework.
Transforming Trauma through an Arts Festival: A Psychosocial Case Study [55].	A two‐day workshop with three integrated parts, as part of The Big Anxiety program in Warwick, Queensland.	Number of participants is not reported. Participants were self‐selecting from the community with a significant number (no figure reported) being First Nations women touched by the loss of children to suicide.	Aim: To outline and describe the theoretical framework and approach of the program to understand how post‐traumatic growth occurred within the program. Methodology: Mixed methods study with focus on phenomenological analysis. The study involved a survey with open ended narrative responses and interviews that were video recorded.	The study reported finding a range of beneficial effects; however, the paper does not focus on these outcomes or describe them in detail. The paper details the process and theoretical basis for the program and recommends similar co‐created creative programs for communities.
At arm's length: A qualitative study of suicide prevention barriers among those experienced with suicide loss [56].	The Australian Capital Territory Coronial Counselling Service (ACTCCS) provides counselling to people affected by a death requiring investigation by the ACT Coroners Court.	Clients of ACTCCS (*n* = 16) who had experienced suicide loss as well as one person who had lost a loved one to suicide but had not engaged with ACTCSS (n = 1).	Aim: Investigate the experience of family, carers and community members impacted by a suicide death in the Australian Capital Territory. Methodology: Qualitative study (semi‐structured interviews) guided by theoretical frameworks of trauma‐informed and restorative practice. An inductive approach to thematic analysis (Framework method) was undertaken.	The study identified three interconnected key themes; disconnected spaces, fragmented, episodic and reactive care and exclusion and marginalisation of families and carers. It was identified that there are significant gaps in the health system for supporting people who experience suicidal distress and their families.
Co‐ideation and co‐design in co‐creation research: Reflections from the ‘Co‐Creating Safe Spaces’ project [57].	Co‐ideation and co‐design of a safe space evaluation (Co‐Creating Safe Spaces project). Safe spaces are trauma‐informed and nonclinical supports involving the use of a peer workforce where people can access support for mental health and suicide‐related distress.	No study population. The co‐ideation and co‐design involved members of safe space steering committees from three services and an Australian suicide prevention organisation. Researchers from Australian universities were also involved.	Aim: To explore the utility of the co‐ideation and co‐design framework using a case study approach. Methodology: Instrumental case study grounded in experience designing and coordinating the Co‐Creating Safe Spaces project. The project involved an evaluation co‐design workshop, a subsequent online survey to capture information on suitable outcome measures.	The case study described that the co‐ideation and co‐design framework did not account for the relational aspect of co‐ models as well as power dynamics and ethical and political aspects. The development of the co‐evaluation was also described.
Aesthetic Enactment: Engagement with Art Evoking Traumatic Loss [58].	A visual matrix following a solo performance lecture and short film at the Big Anxiety Festival in Sydney, New South Wales.	Number of participants is not reported. Participants were attendees at the Big Anxiety Festival in Sydney. No personal information was gathered; however, some participants reported that they had experience of family suicide.	Aim: To assess whether sensory affective expression in a supportive group context, enables audience to engage with their experiences. Methodology: Qualitative study using self‐reflexive researcher observation and interpretation, building on interpretive frame set by participants.	Participants responded to the session imagistically and metaphorically. The study concluded that participants enacted what they had viewed in an embodied aesthetic mode that facilitates engagement and generativity in the subject matter.
Promoting Engagement With Smartphone Apps for Suicidal Ideation in Young People: Development of an Adjunctive Strategy Using a Lived Experience Participatory Design Approach [59].	LifeBuoy is a smartphone app designed to help young people manage suicidal thoughts through distress tolerance and emotional regulation. The approach is grounded in dialectical behaviour therapy and acceptance and commitment therapy.	Young people who participated in the LifeBuoy trial were interviewed for the study (*n* = 16). From this group, three were invited and participated in a further two workshops. Participants reported as having previously experienced suicidal thoughts.	Aim: To describe how user‐focused design can be applied to increase engagement with digital mental health interventions. Methodology: Qualitative study using interviews and workshops to design and refine a prototype for an app promotion and engagement strategy.	The paper describes the process undertaken by the study to develop the strategy. The study identified that brief, practical and inspirational content, delivered over social media (Instagram), may motivate young people to engage with the intervention. In depth information was found to be suitable for delivery via a blog written by clinicians and people with lived experience.
Peer Intervention following Suicide‐Related Emergency Department Presentation: Evaluation of the PAUSE Pilot Program [60].	PAUSE was developed and implemented by lived experience organisation Brook RED, supporting people following presentation at the emergency department with suicidal ideation, suicide attempt or an episode of self‐injury. In the program, peer workers provide support for up to 13 weeks.	People who presented to the Logan General Hospital Emergency Department were invited to engage with PAUSE. Of these referrals, evaluation data was collected for 54 participants. A further ten participants were also interviewed.	Aim: To evaluate the effectiveness and acceptability of PAUSE to reduce suicidality and mental illness symptoms and increase hope following suicide‐related hospital presentation. The study also sought to understand key factors contributing to the impact or outcomes of the program. Methodology: Co‐designed with peer workers, a mixed methods approach was used. This involved a questionnaire with the General Health Questionnaire Suicide Scale, Adult Hope Scale and Kessler Psychological Distress Scale (K10), PAUSE Experience Questionnaire and Semi‐Structured Interviews.	The study identified four themes as critical factors underlying the programs effect; holistic and responsive support, peer workers understanding participants' experiences, treating the participants like people rather than clients and ongoing social connectedness. Participants reported that the program was beneficial and suicidal ideation and hope scores improved after the program. The study reported that the PAUSE peer support program was an acceptable and effective model of care following suicide‐related hospital presentations.
Lived experience perspectives guiding improvements to the Systematic Tailored Assessment for Responding to Suicidality protocol [61].	STARS‐p uses as structured professional judgement approach to assessing suicidal states, psycho‐social risk and protective factors to inform client care plans.	Participants (*n* = 33) were those who has a lived experience of their own suicidality, surviving a suicide attempt, losing a loved one to suicide or caring for someone in suicidal distress.	Aim: To explore the perceptions of those with lived experience regarding the STARS‐p and elicit suggestions for improvement of wording and language in the tool. Methodology: Qualitative descriptive design using an interactive 3.5 h workshop hosted by Roses in the Ocean as part of the National Lived Experience Summit, 2021.	Three categories of meaning were elicited; STARS philosophy, what STARS aspires to and continuity of care and meeting needs. Improvements were suggested including additions, rewording and reordering of content. The findings will inform a redesign process for the next edition of the STARS‐p.
Informing and Sustaining Participation of Lived Experience in the Suicide Prevention Workforce [62].	Exploratory research on the lived experience workforce in suicide prevention.	Purposive sampling was used to recruit participants who had been in the lived experience workforce for at least 12 months (*n* = 13). Participants had experienced their own suicidality, were bereaved by suicide, were a carer for someone who has been suicidal or described having more than one of these experiences. They had also completed training from lived experience organisation Roses in the Ocean.	Aim: To explore the experiences of the lived experience workforce and identify issues critical to continued participation in the suicide prevention lived experience workforce. Methodology: Semi‐structured interviews were conducted with participants.	Five themes were identified; support, passion, personal impact of lived experience workforce, training, and work diversity within the lived experience workforce.
A qualitative analysis of self‐reported suicide gatekeeper competencies and behaviour within the Australian construction industry [63].	Bluehats Suicide Prevention Program provides suicide prevention activities across a continuum of support.	Participants were 22 ‘Bluehats’ from various roles within the workplace. 12 (55%) had lived experience of self‐harm or suicidal behaviour.	Aim: To understand the impact of the Bluehats' training on competencies and how these translated into suicide prevention behaviour. Methodology: Semi‐structured interviews were conducted and data analysed using a deductive orientation to thematic analysis.	High levels of motivation and capability were reported by participants. These were due to lived experience, training, satisfaction from helping others and an environment wherein they felt valued. An increase in capability and motivation was reported following training.
Evaluation of a New Online Program for Children Bereaved by Suicide: The Views of Children, Parents, and Facilitators [64].	The Let's Talk Suicide program is a two‐week online program for children and their families providing support and services to children and their parents after the suicide of a parent or sibling.	Participants were parents (*n* = 7), children (*n* = 4) and facilitators (*n* = 3) from the program. Where reported, the death by suicide was either a mother or father, or brother and occurred between five and ten years before participation.	Aim: To evaluate participant and facilitator experiences and the perceived helpfulness of the Let's Talk Suicide program. Methodology: Semi‐structured interviews were conducted and reporting in alignment with the Consolidated Criteria for Reporting Qualitative Research.	Four major themes were elicited; the importance of suicide‐bereavement specific support, appraising the online environment, expectations and outcomes of the program for participants and parents, and parents' experience of involvement in the program. Overall, both children/adolescents and parents were satisfied with the program.
Active involvement of people with lived experience of suicide in suicide research: a Delphi consensus study [15].	Not applicable, Delphi consensus study.	Panels in the study were comprised of people with lived experience of suicide (*n* = 44) and suicide researchers (*n* = 29) of which 14 identified as having lived experience of suicide.	Aim: To develop guidelines on active involvement of people with lived experience of suicide in research. Methodology: Delphi consensus method using two expert panels and three rounds of the survey. Statements were obtained from the literature and qualitative interviews undertaken in a recent‐related study conducted by the researchers.	A total of 96 statements were endorsed by both panels across 17 domains. The statements provide a guide for researchers and lived experience on research collaboration and coproduction.
Which programmes and policies across health and community settings will generate the most significant impacts for youth suicide prevention in Australia and the UK? Protocol for a systems modelling and simulation study [65].	SEYMOUR (System Dynamics Modelling for Suicide Prevention) is a proposed project to develop a system dynamics model to inform youth suicide prevention policy, planning and implementation in Australia and the UK.	Young people aged 12–25 years with lived/living experience of self‐harm and/or suicidal behaviour living in the catchment area of North‐West Melbourne or Birmingham.	Aim: The proposed study aims to develop and validate a model to inform a suicide prevention intervention, develop an implementation strategy of the model and adapt and validate the model to the UK context. Methodology: The proposed study will adopt a comparative case study design. It is a mixed‐methods study delivered by three inter‐related work packages guided by Forrester's SDM framework.	Not available. Paper is a research protocol.
#chatsafe 2.0. updated guidelines to support young people to communicate safely online about self‐harm and suicide: A Delphi expert consensus study [66].	Not applicable, Delphi consensus study.	Young people aged between 15–25 years who has seen, communicated about or wanted to communicate online about self‐harm or suicide.	Aim: To update and expand the #chatsafe guidelines to better reflect the evidence and ways that young people use social media to communicate about suicide, and to include guidance on self‐harm. Methodology: Delphi consensus method using a youth panel and professional panel over two rounds of questionnaires. Six round table consultations were also conducted to inform the Delphi study.	A total of 191 items were endorsed by both panels to be included in the final guidelines. Final guidelines were organised into eight sections; general tips, creating self‐harm and suicide content, consuming self‐harm and suicide content, livestreams of self‐harm and suicide acts, self‐harm and suicide games, pacts and hoaxes, self‐harm and suicide communities, bereavement and communicating about someone who has died by suicide and guidance for influences.
Safety, Acceptability, and Initial Effectiveness of a Novel Digital Suicide Prevention Campaign Challenging Perceived Burdensomeness [67].	Better Off With You, digital suicide campaign underpinned by the Interpersonal Theory of Suicide.	Participants were between 20–65 years of age, had reliable mobile internet and not have had a specific plan, or attempted to end their life within the last 6 months (Survey, *n* = 157; Interview, *n* = 15). Some participants reported having various types of lived experiences (personally experiencing suicidality or having someone close to them experience suicidality including dying by suicide).	Aim: To test the real‐world experience of seeing a digital suicide prevention campaign via staggered exposure to content, and via viewing on a mobile device. Methodology: Mixed methods pilot study in two targeted Australian communities. Surveys were collected at baseline and after 1‐week exposure to the campaign videos and website. Qualitative interviews were then conducted with a subset of participants.	Participants rated the campaign as highly engaging and relevant to local communities. They reported the campaign as unique, safe and impactful. Exposure to the campaign did not result in any changes to perceived burdensomeness, psychological distress or help‐seeking (outcomes of interest). The study concluded the campaign was safe for release into the wider community.

**Table 5:** Key lived experience participation descriptors of included studies.

Title (Author, Year)	Lived experience definition	Participant lived experience	Recruitment strategy	Suicide‐related exclusion criteria	Role of people with lived experience	Safety measures
Confidence and attitudes of pharmacy students towards suicidal crises: patient simulation using people with a lived experience [36].	Not defined	Lived experience not reported	Through organisation or service	Not identified	Intervention activity	Not described
Ethical Issues to Consider in Designing Suicide Prevention Studies: An Expert Consensus Study [14].	Roses in the Ocean's definition; people with personal experience of suicidal thoughts, surviving a suicide attempt, having cared for someone through a suicidal crisis, or been bereaved by suicide	Specific lived experience types not reported.	Through organisation or service	Suicide attempt or bereavement less than 6 months ago	Participants, research conduct	Not described
Acceptability, Safety, and Resonance of the Pilot Digital Suicide Prevention Campaign ‘Better Off With You’: Qualitative Study [37].	Specific type of lived experience; people who have experienced suicidal thoughts and actions	More than one type of lived experience combined	Through organisation or service, social media, through past research activities	Suicide attempt or ‘seriously contemplated suicide’ less than 12 months ago	Participants	Wellness or readiness plan, clinician present or available, adherence to guidelines or specific practice, crisis service contacts or referral, follow up contact including email or call
A survey of people with lived experience of suicide‐related behaviour in Queensland, Australia: Their experiences with available resources [32].	Own definition; Individuals who are affected by suicide‐related behaviour (defined as suicide, suicide attempt, or self‐harm where intent is unclear), such as people who attempt suicide, are bereaved by suicide, and who care for people in these groups,	Bereaved, cared for someone who had attempted or were bereaved by suicide, have attempted suicide	Through organisation or service, social media, through a network or register	Not identified	Participants, research advisory or advice in an informal way	Not described
Crafting safe and effective suicide prevention media messages: outcomes from a workshop in Australia [38].	Specific type of lived experience; included people who had been suicidal and/or were bereaved by suicide	Being suicidal and/or being bereaved by suicide.	Attendance at a specific event or workshop	Not identified	Participants	Not described
Use of Web Conferencing Technology for Conducting Online Focus Groups Among Young People With Lived Experience of Suicidal Thoughts: Mixed Methods Research [39].	Specific type of lived experience; lived experience of suicidal thoughts/suicidal ideation	Having experienced suicidal ideation	Through organisation or service, social media	Suicide attempt within the last month or thoughts of suicide within the last 2 weeks.	Participants	Clinician present or available, follow up contact including email or call, check in at end of involvement, identification of risk before or during involvement, risk management log, contact support on behalf of participant
Preliminary Evaluation of Lived Experience of Suicide Training: Short‐, Medium‐ and Longer‐Term Impacts of Our Voices in Action Training [40].	Roses in the Ocean's definition; people with personal experience of suicidal thoughts, surviving a suicide attempt, having cared for someone through a suicidal crisis, or been bereaved by suicide	Multiple types of experiences combined, being bereaved by suicide, having suicidal thoughts, cared or continuing to care for someone who is suicidal or attempted suicide and having attempted suicide	Attendance at a specific event or workshop	Not identified	Intervention activity, participants	Wellness or readiness plan
Development of best practice guidelines for suicide‐related crisis response and aftercare in the emergency department or other acute settings: a Delphi expert consensus study [41].	Definition unclear	Having personal experience of suicidal behaviour, being a carer of someone with suicidal behaviour and having both types of experience combined	Through organisation or service	Not identified	Participants	Not described
Perspectives of rural health and human service practitioners following suicide prevention training programme in Australia: A thematic analysis [42].	Suicide Prevention Australia's definition; having experienced suicidal thoughts, survived a suicide attempt, cared for someone who has attempted suicide, been bereaved by suicide, or been touched by suicide in another way.	Lived experience not reported	Not described	Not identified	Intervention activity	Clinician present or available
A Mobile Text Message Intervention to Reduce Repeat Suicidal Episodes: Design and Development of Reconnecting after a Suicide Attempt (RAFT) [43].	Specific type of lived experience; having a history of suicide attempt.	Having a history of a suicide attempt for one participant group. Other group lived experience composition is not reported	Through organisation or service, social media, through a network or register	Suicide attempt less than one month ago and current severe suicidal ideation (if a current suicide plan, means, or intent was endorsed).	Participants	Not described
Developing best practice guidelines for the psychosocial assessment of Aboriginal and Torres Strait Islander people presenting to hospital with self‐harm and suicidal thoughts [44].	Definition unclear	Lived experience not reported	Through organisation or service, advisors/knowledge of research team	Not identified	Participants	Not described
Involving mental health service users in suicide‐related research: A qualitative enquiry model [45].	Specific type of lived experience; service users undergoing suicidal crises	Lived experience not reported	Through organisation or service	Not identified	Participants	Check in at end of involvement
Carers' motivations for, and experiences of, participating in suicide research [4].	Specific type of lived experience; a person caring for someone who has previously attempted suicide	Carers of children, friends and partners who had previously attempted suicide	Through organisation or service, social media, through a network or register	Not currently at risk of suicide	Participants	Crisis service contacts or referral
Assessing students' mental health crisis skills via consumers with lived experience: a qualitative evaluation [46].	Definition unclear	Lived experience not reported	Not described	Not identified	Intervention activity, participants	Not described
Drafting the Aboriginal and Islander Mental Health Initiative for Youth (AIMhi‐Y) App: Results of a formative mixed methods study [47].	Not defined	Bereavement as a result of death by suicide and current suicidal ideation.	Through organisation or service, social media, through a network or register	Not identified	Participants, research conduct	Crisis service contacts or referral, follow up contact including email or call
Consumer and carer perspectives of a zero suicide prevention program: A qualitative study [48].	Specific type of lived experience; Consumers refers to healthcare patients who have experienced a recent suicide attempt that required intervention at the GCHHS, and carers refers to a close personal contact of the consumer involved in their intake and recovery	People who had a recent suicide attempt and their carers.	Not described	Not identified	Participants	Not described
A lived experience co‐designed study protocol for a randomised control trial: the Attempted Suicide Short Intervention Program (ASSIP) or Brief Cognitive Behavioural Therapy as additional interventions after a suicide attempt compared to a standard Suicide Prevention Pathway (SPP) [68].	Specific type of lived experience; Lived experience of mental health and people who attempt suicide or experience suicidality after a suicide attempt	Specific lived experience types not reported.	Through organisation or service	Not identified	Research advisory or advice in an informal way	Not described
Taking the next step: A qualitative study examining processes of change in a suicide prevention program incorporating peer‐workers [17].	Not defined	Specific lived experience types not reported.	Through organisation or service	Not identified	Participants, research conduct	Not described
Developing an Intervention for Suicide Prevention: A Rapid Review of Lived Experience Involvement [3].	Roses in the Ocean's definition; people with personal experience of suicidal thoughts, surviving a suicide attempt, having cared for someone through a suicidal crisis, or been bereaved by suicide	Not applicable, rapid review	Not applicable, rapid review	Not applicable, rapid review	No role identified	
How is participating in suicide prevention activities experienced by those with lived and living experiences of suicide in Australia? A qualitative study [2].	Own definition; those who have survived their own suicide attempt, been bereaved by the death of someone who died by suicide or supported someone who was/is suicidal.	Bereaved by suicide or survived a suicide attempt	Through organisation or service	Not identified	Participants, research conduct, research advisory or advice in an informal way, funding	Not described
Ethical and political implications of the turn to stories in suicide prevention [49].	Not defined	Not applicable, philosophical discussion paper	Not applicable, philosophical discussion paper	Not applicable, philosophical discussion paper	No role identified	Not described
Developing a Suicide Prevention Social Media Campaign With Young People (The #Chatsafe Project): co‐design Approach [13].	. Specific type of lived experience; having suicide ideation or supporting a friend through suicide ideation and lost someone to suicide	Supporting a friend experiencing suicidal ideation, having suicidal ideation or losing someone close to suicide	Through organisation or service, social media, attendance at specific event or workshop	Not identified	Intervention activity, participants	Wellness or readiness plan, clinician present or available, risk management log, room agreement
Developing a post‐discharge suicide prevention intervention for children and young people: a qualitative study of integrating the lived‐experience of young people, their carers, and mental health clinicians [50].	Roses in the Ocean's definition; people with personal experience of suicidal thoughts, surviving a suicide attempt, having cared for someone through a suicidal crisis, or been bereaved by suicide	Suicide attempt or self‐harm incident or caring for a young person following a suicide attempt or self‐harm incident	Through organisation or service, through a network or register	Not identified	Participants	Clinician present or available
Exploring Community‐Based Suicide Prevention in the Context of Rural Australia: A Qualitative Study [51].	Roses in the Ocean's definition; people with personal experience of suicidal thoughts, surviving a suicide attempt, having cared for someone through a suicidal crisis, or been bereaved by suicide	Specific lived experience types not reported.	Advisors/knowledge of research team, Google searches to identify relevant people	Not identified	Participants	Not described
Stakeholder insights into implementing a systems‐based suicide prevention program in regional and rural Tasmanian communities [52].	Roses in the Ocean's definition; people with personal experience of suicidal thoughts, surviving a suicide attempt, having cared for someone through a suicidal crisis, or been bereaved by suicide	Specific lived experience types not reported.	Not described	Not identified	Participants	Not described
A model of lived experience leadership for transformative systems change: Activating Lived Experience Leadership (ALEL) project [5].	Definition unclear	Specific lived experience types not reported.	Through organisation or service	Not identified	Participants, research conduct, research advisory or advice in an informal way	Not described
Understanding and detecting behaviours before a suicide attempt: A mixed‐methods study [53].	Not defined	Specific lived experience types not reported.	Not described	Not identified	Participants	Not described
Co‐creation of new knowledge: Good fortune or good management? [54].	Not defined	Specific lived experience types not reported.	Not described	Not identified	Participants, research conduct	Not described
Transforming Trauma through an Arts Festival: A Psychosocial Case Study [55].	Specific type of lived experience; touched by the loss of children to suicide	Loss of children to suicide	Not described	Not identified	Intervention activity, participants	Clinician present or available, provided questions before interview
At arm's length: A qualitative study of suicide prevention barriers among those experienced with suicide loss [56].	Specific type of lived experience; experienced suicide loss includes parents, grandparents, spouse of partner, aunt of uncle, sibling, children and school staff	Loss of loved one to suicide	Through organisation or service	Not identified	Participants	Adherence to guidelines or specific practice
Co‐ideation and co‐design in co‐creation research: Reflections from the ‘Co‐Creating Safe Spaces’ project [57].	Definition unclear	Lived experience involvement reported generally with some participants listed as having experienced emotional distress and/or suicidal crisis	Through organisation or service, through a network or register, advisors/knowledge of research team	Not identified	Intervention activity, participants, research conduct	Not described
Aesthetic Enactment: Engagement with Art Evoking Traumatic Loss [58].	Specific type of lived experience; experience of family suicide and loss to suicide	Family suicide and loss to suicide	Not described	Not identified	Intervention activity, participants	Clinician present or available, facilitator/researcher training/qualifications
Promoting Engagement With Smartphone Apps for Suicidal Ideation in Young People: Development of an Adjunctive Strategy Using a Lived Experience Participatory Design Approach [59].	Specific type of lived experience; a history of attempted suicide	History of suicide attempt	Through past research activities	Not identified	Participants	Clinician present or available, crisis service contacts or referral, follow up contact including email or call, identification of risk before or during involvement, facilitator/researcher training/qualifications
Peer Intervention following Suicide‐Related Emergency Department Presentation: Evaluation of the PAUSE Pilot Program [60].	Specific type of lived experience; a personal experience of own mental ill‐health, suicidality, and/or the mental health system	More than one type of lived experience combined	Through organisation or service	Not identified	Intervention activity, participants, research conduct	Crisis service contacts or referral, follow up contact including email or call, facilitator/researcher training/qualifications
Lived experience perspectives guiding improvements to the Systematic Tailored Assessment for Responding to Suicidality protocol [61].	Roses in the Ocean's definition; people with personal experience of suicidal thoughts, surviving a suicide attempt, having cared for someone through a suicidal crisis, or been bereaved by suicide	Own suicidality, survived a suicide attempt, lost a loved one to suicide, or cared for someone in suicidal distress	Attendance at a specific event or workshop	Not identified	Participants	Check in at end of involvement, peer support available at the time of study
Informing and Sustaining Participation of Lived Experience in the Suicide Prevention Workforce [62].	Roses in the Ocean's definition; people with personal experience of suicidal thoughts, surviving a suicide attempt, having cared for someone through a suicidal crisis, or been bereaved by suicide	Own suicidality, being bereaved by suicide, and being a carer for someone who has been or is suicidal. Some participants had more than one experience.	Through organisation or service, attendance at specific event or workshop	Not identified	Participants, research conduct	Facilitator/researcher training/qualifications
A qualitative analysis of self‐reported suicide gatekeeper competencies and behaviour within the Australian construction industry [63].	Definition unclear	Lived experience of self‐harm or suicidal behaviour	Attendance at a specific event or workshop	Not identified	Participants	Identification of risk before or during involvement
Evaluation of a New Online Program for Children Bereaved by Suicide: The Views of Children, Parents, and Facilitators [64].	Specific type of lived experience; bereavement; suicide of a parent or sibling	Suicide bereavement	Attendance at a specific event or workshop	Suicide bereavement less than 6 months ago	Intervention activity, participants	Facilitator/researcher training/qualifications
Active involvement of people with lived experience of suicide in suicide research: a Delphi consensus study [15].	Roses in the Ocean's definition; people with personal experience of suicidal thoughts, surviving a suicide attempt, having cared for someone through a suicidal crisis, or been bereaved by suicide	Specific lived experience types not reported.	Through organisation or service, social media, through past research activities	Suicide bereavement less than 6 months ago or suicide attempt less than 6 months ago	Participants, research conduct	Not described
Which programmes and policies across health and community settings will generate the most significant impacts for youth suicide prevention in Australia and the UK? Protocol for a systems modelling and simulation study [65].	Specific type of lived experience; suicidal behaviour	Lived/living experience of self‐harm and/or suicidal behaviour and family members/carers of those with lived experience of self‐harm and/or suicidal behaviour	Through organisation or service	Not identified	Participants, research advisory or advice in an informal way	Facilitator/researcher training/qualifications
#chatsafe 2.0. updated guidelines to support young people to communicate safely online about self‐harm and suicide: A Delphi expert consensus study [66].	Definition unclear	Specific lived experience types not reported.	Social media	Not identified	Participants	Not described
Safety, Acceptability, and Initial Effectiveness of a Novel Digital Suicide Prevention Campaign Challenging Perceived Burdensomeness [67].	Definition unclear	Thought about ending own life, considered acting on thoughts of suicide, lost someone close to suicide, someone close to them has attempted to end their life, someone close to them has or was having thoughts of ending their life.	Through organisation or service	Specific plan or attempted to end life within last 6 months.	Intervention activity, participants	Follow‐up contact including email or call, check in at end of involvement

### 3.2 Key Definitions of Lived Experience

Lived experience was not consistently defined across the studies with only 12 of the 42 studies making a statement that defined lived experience (e.g. we define lived experience as…). Definitions promulgated by two suicide prevention organisations were stated in some studies, namely Roses in the Ocean and Suicide Prevention Australia. When lived experience type is identified for study participants, studies most frequently engage those who are bereaved (12 studies). Lived experience identifiers can be found at Table 5.

### 3.3 Pathways in and out of Participation of Suicide Prevention

The main recruitment strategy used is obtaining participants through an existing organisation or service (24 studies). Recruitment strategies are listed in Table 5. Most studies did not describe any specific requirements of people with lived experience before participating in the research, except one study where people with lived experience were asked to prepare a wellness plan [37] and another study requiring people with lived experience to undertake a readiness exploration process [40].

When it came to exclusion based on recency of experience, six studies excluded people with lived experience for recent suicide attempt, three studies for recent bereavement and four studies for recent thoughts of suicide. Time periods given were consistently ‘6 months ago’ for bereavement but inconsistent for recent suicide attempt or thoughts of suicide with periods ranging from ‘not currently’ [43] to ‘more than 12 months ago’ [37]. Exclusion criteria is listed in Table 5.

### 3.4 Specific Roles of People With Lived Experience Within the Included Studies

When discussing the role of people with lived experience, almost all studies referred to people with lived experience as research participants (37 studies) with only five not involving people with lived experience as study participants. Smaller numbers of studies included people with lived experience in the conduct of the research (10 studies), in the delivery of an intervention (11 studies) or to provide advice on research activities or findings (five studies). Two studies [3, 49] did not report including people with lived experience at all, rather they described ethical considerations when including people with lived experience and how people with lived experience are included in the development of suicide interventions respectively. One study [2] engaged a lived experience organisation as a funding partner. Less than half of these studies involved people with lived experience in more than one of these ways in the same project (19 studies). The role of people with lived experience in each study is listed in Table 5.

### 3.5 Impacts From Involvement and Safety Measures Employed

Only nine studies reported any impact of participation on people with lived experience. Four studies reported positive impacts for people with lived experience; however, the positive impact was only described for participation in the intervention, not the research activity itself [17, 46, 55, 60]. One study [4] reported that a few participants experienced negative but manageable emotions, and two studies reported both positive and negative impacts from participating in the research [13, 58]. One study [59] reported ‘no negative’ impacts and a further study [67] reported ‘no impact’. Both the rapid review [3], and the critical discussion [49] did not mention impacts on people with lived experience, although both papers did not appear to include people with lived experience.

When looking at the impact of involving people with lived experience on others involved in the research, only one study reported benefits to participants of the intervention who were not in a lived experience‐related role [46]. No studies reported on any impacts on the research team when involving people with lived experience of suicide in research.

Almost half of the studies (20 studies) did not report any safety measures in place for participants. There were 10 studies (of 22) who employed more than one safety strategy with the most being six safety measures [39]. Specific safety measures reported on are listed in Table 5.

## 4 Discussion

The scoping review aimed to describe the current literature base as it relates to lived experience inclusion and participation in suicide prevention identifying four themes from the analysis: (1) key definitions of lived experience inclusion, (2) pathways in and out of participation of suicide prevention, (3) specific roles of people with lived experience, and (4) impacts from involvement and safety measures employed.

The scoping review did not locate any papers on lived experience inclusion published before 2016. This is consistent with the results from an international rapid review, which found that most papers on this topic were published after 2015 [3]. There was a sharp increase in research involving people with lived experience in 2020, followed by a decline to pre‐2020 levels in 2022, and then a significant increase in 2023. This may be a result of increased attention to lived experience‐related topics as part of the Prime Minister's Advice into suicide prevention [1]. It also may be reflective of the emergence of lived experience as a concept in research and/or practice. To the awareness of the research team, there has been no review into the emergence of lived experience as a concept or term in suicide prevention. Such a review would add to the documented history of the inclusion of people with direct experience.

It has been posited that the field of suicidology (suicide‐related research) has traditionally limited itself to positivist explanations of suicide, favouring quantitative approaches over person‐centred knowledge generation [69]. The recency of papers included in this review may reflect changing attitudes towards experiential knowledge. It may be suggested that the study of suicide is increasingly turning to qualitative methods with some arguing that qualitative methods will move suicidology to a place of *understanding* rather than *explanation* of suicide [70]. The current study did not uncover a clear pattern or changes in methods employed over time, although mixed methods studies and thematic analysis were identified as common. This may indicate a lack of consensus on best research methodologies for lived experience‐related topics. It is of interest that in 2023, there was a broad range of analytic methods employed, with more collaborative aims including systems dynamic modelling, user focussed design, co‐design, visual matrix, phenomenology and iterative and shared analysis.

There are definitional challenges when it comes to identifying who is included. Many studies did not define or state‐specific descriptors relating to ‘lived experience’. However, there appeared to be a preference for involving people with carer or bereavement perspectives as study participants over those who had experienced suicide states themselves. Definitional challenges not only impact retrieval of literature where alternate terms may be used or terms used inconsistently but also generate uncertainty with the exact parameters for who has been included. Recently there has been a growing movement in critical suicide studies where these definitional challenges are discussed as continuously evolving [71]. Definitional challenges in suicide prevention are not new and have been studied and reported under the International Study of Definitions of English‐Language Terms for Suicidal Behaviours [27], whilst noting that defining lived experience was not a feature of this study.

The review identified a lack of reporting of lived experience inclusion on research teams. This lack of reporting may not reflect a lack of inclusion, but rather a lack of disclosure. Research related to nondisclosure of lived experience of suicide in workplaces has been identified as a clear gap in the literature [72]. In their Delphi study on lived experience involvement in suicide research, Krysinska, Ozols, et al. [15] reported that just under half of the people identifying as researchers also reported having lived experience. It was not reported whether this lived experience was disclosed in their work. Research on suicide disclosure highlights the need to allow individual choice on disclosure. In fields such as social work, pressure to disclose lived experience may lead a researcher to feel fear of stigmatisation and a lack of safety [73]. It is also possible that the boundary between personal/professional is unclear in the research context as it has been explored elsewhere in peer work contexts [74]. Other authors argue that while lived experience may exist and be undisclosed, to be a representative lived experience voice requires being ‘out’ with disclosure [5]. This has implications for directives such as that by the Lancet Psychiatry to disclose lived experience involvement in research requesting publication [12]. Researchers are encouraged to consider and report on the ways people with lived experience are involved in their work. Researchers are also encouraged to consider how people with lived experience might become involved in the conduct of research or delivery/design of an intervention.

Researchers, and the ethical review boards to which they are accountable, can act as key gatekeepers by using inclusion and exclusion criteria to determine who participates in the generation of knowledge [75]. The review found that these criteria are inconsistently applied in suicide research and that studies rarely explained their reasoning, particularly regarding specific time periods since the suicide experience. These time periods vary across studies and rely on self‐disclosure of the suicide experience by participants. Studies without recency exclusion criteria are not more likely to report adverse outcomes, although it warrants noting that most studies did not report on the impact of involvement. One study identified that five individuals were excluded from the study based on current thoughts of suicide or a suicide attempt in the previous month, yet it was not described how these participants were managed following exclusion from the research [43]. Recent research captured the perspectives of those with lived experience and reported that exclusion based on acute symptoms is perceived as related to stigmatised beliefs about the capacity of people with lived experience [76]. Further, work has been done to standardise distress management in sensitive research such as the Qualitative Research Distress Protocol [77]. Approaches like this, if tested for effectiveness, may offer a standard and appropriate way for managing distress over excluding those who may be viewed as a risk in research activities.

The review mapped specific roles people with lived experience held in the research process. While this study excluded papers where people with lived experience were included for knowledge generation of the suicide experience, most papers still only included people with lived experience as participants or did not document the involvement of people with lived experience in the conduct of the research or intervention. Further, most studies involved people with lived experience in only one capacity, with a small number of studies reporting lived experience involvement in multiple roles. The role of people with lived experience has evolved, with recent studies involving them in research conduct, advisory roles, or as members of the intervention activity (e.g., [60]). More recent studies are also likely to involve people with lived experience in multiple ways such as advisors on a research steering committee and in the conduct of the research. These shifts in inclusion, with a move to deeper involvement of people with lived experience, can be seen in broader mental health research with more detailed specification of how and why people with lived experience can be included and lead research [78].

Understanding the impact of involvement on both people with lived experience and those without is important. While some studies did report on the impacts of participation for people with lived experience, only one study addressed the impact on those without lived experience [46]. This study included people with lived experience as part of the intervention and participants in the study (pharmacy students) were reported to have benefited from the involvement of people with lived experience. This apparent disconnect in safety needs between people with lived experience and suicide researchers emerged in the Delphi consensus study on ethical issues to consider in designing suicide prevention studies [14]. Given the rise in discussions on researcher wellbeing for topics such as death and dying (e.g., [79]), greater attention to the impact on all involved in the research journey should be afforded.

## 5 Limitations

The review cast a wide net to capture research that directly described lived experience engagement and studies that included people with lived experience for suicide prevention aims. Because of this, it is possible that many included studies did not see the importance of reporting elements of the involvement of people with lived experience. Details that were missing or poorly defined include definitions or boundaries of what lived experience was, as it related to the study, details around safety and support for those involved in the research team and whether there was any impact from participating in the activity for people with lived experience or others broadly.

There can be argument that the terminology used to identify people who have direct experience of suicide has changed through time. This limits the sensitivity of this scoping review in identifying papers that have included people with lived experience but have used different terminology (such as patient and consumer). This review should not be taken as a historical account of the inclusion of people with direct experience of suicide, but rather an analysis of their involvement under the label of ‘people with lived experience of suicide’.

The scoping review methodology is also not without limitation. As the approach is designed to be exploratory in nature, it does not capture the depth of the literature base [80]. This means that concrete themes are less able to be identified. The current study should be considered as a broad look at the literature base to describe the extent, range and nature [28] of literature describing lived experience participation in suicide prevention.

While this review did not describe the presence of resources in the grey literature, there is some evidence that the practicalities of involving people with lived experience of suicide are commonly reported in the grey literature, particularly around frameworks for participation [22] and principles for engaging with people with lived experience of suicide [8]. Expansion of this review to examine grey literature may provide a more holistic picture on the ‘how’ of lived experience inclusion.

Due to the consensus obtained in screening, the two updated searches were screened by two reviewers for title and abstract, but only one reviewer for the full text. This may have proved a limitation in the review.

## 6 Key Messages

There are a number of practical considerations arising from this review that may be of relevance to researchers and policy makers. To develop the literature base, consistency in reporting is needed. This includes agreement or specification of assumed terminology and standardisation in reporting the ‘how’ of inclusion. As the inclusion of people with lived experience appears to be growing, identifying the most appropriate mechanisms for inclusion is important to reduce concerns over safety which result in exclusion of certain groups with lived experience. Improving reporting on lived experience inclusion will assist building a literature base from which appropriate standard practice can be developed.

## 7 Conclusions

While there is acknowledgement that involving people with lived experience in suicide prevention activities is important [1] and beneficial [2], there is a lack of scientific evidence on how to best go about this. This scoping review has built on previous efforts at mapping inclusion [3, 25] through an expanded focus on inclusion in suicide prevention broadly. The review identified a changing landscape of research activities including people with lived experience of suicide with an increase in more recent years. Despite the growing evidence base, definitional challenges and safety‐related inconsistencies were identified. It was also found that participation as research subjects is a primary way people with lived experience of suicide are included in suicide prevention and research activities; however, there is some indication that this is changing with the increase of more qualitative or collaborative research methods. With many gaps still existing in describing how lived experience is included in suicide prevention activities, researchers are encouraged to make any inclusion explicit in their research publications and translation activities.

## Author Contributions

**Hayley Purdon:** conceptualisation, investigation, funding acquisition, writing – original draft, methodology, writing – review and editing, formal analysis, project administration, data curation. **Tania Pearce:** methodology, writing – review and editing, validation. **Bess Jackson:** writing – review and editing, validation. **Sarah Wayland:** writing – review and editing, validation, supervision, methodology. **Myfanwy Maple:** supervision, writing – review and editing, methodology.

## Ethics Statement

The authors have nothing to report.

## Consent

The authors have nothing to report.

## Conflicts of Interest

The authors declare no conflicts of interest.

## Permission to Reproduce Material from Other Sources

The authors have nothing to report.

## Data Availability

The data that support the findings of this study are available from the corresponding author upon reasonable request.
